# Using a theory of change to develop an integrated intervention for depression, diabetes and hypertension in Zimbabwe: lessons from the Friendship Bench project

**DOI:** 10.1186/s12913-021-06957-5

**Published:** 2021-09-07

**Authors:** Tiny Tinashe Kamvura, Jean Turner, Ephraim Chiriseri, Jermaine Dambi, Ruth Verhey, Dixon Chibanda

**Affiliations:** 1 Research Support Centre, Faculty of Medicine and Health Sciences, University of Zimbabwe, Harare, Zimbabwe; 2 Rehabilitation Sciences Unit, Faculty of Medicine and Health Sciences, University of Zimbabwe, Harare, Zimbabwe

## Abstract

**Background:**

Non-communicable diseases (NCDs) are projected to become the leading cause of disability and mortality in sub-Saharan Africa by 2030; a vast treatment gap exists. There is a dearth of knowledge on developing evidence-based interventions that address comorbid NCDs using a task-shifting approach. The Friendship Bench, a brief psychological intervention for common mental disorders delivered by trained community grandmothers, is a promising intervention for comorbid NCDs. Although task-shifting appears to be a rational approach, evidence suggests that it may bring about tension between existing professionals from whom tasks are shifted. A Theory of Change approach is an effective way of managing the unintended tension by bringing together different stakeholders involved to build consensus on how to task shift appropriately to the parties involved. We aimed to use a theory of change approach to formulating a road map on how to successfully integrate diabetes and hypertension care into the existing Friendship Bench in order to come up with an integrated care package for depression, hypertension and diabetes aimed at strengthening NCD care in primary health care systems in Zimbabwe.

**Method:**

A theory of change workshop with 18 stakeholders from diverse backgrounds was carried out in February 2020. Participants included grandmothers working on the Friendship Bench project (*n* = 4), policymakers from the ministry of health (*n* = 2), people with lived experience for the three NCDs (*n* = 4), health care workers (*n* = 2), and traditional healers (*n* = 2).

Findings from earlier work (situational analysis, desk review, FGDs and clinic-based surveys) on the three NCDs were shared before starting the ToC. A facilitator with previous experience running ToCs led the workshop and facilitated the co-production of the ToC map. Through an iterative process, consensus between the 18 stakeholders was reached, and a causal pathway leading to developing a framework for an intervention was formulated.

**Results:**

The ToC singled out the need to use expert clients (people with lived experience) to promote a patient-centred care approach that would leverage the existing Friendship Bench approach. In the face of COVID-19, the stakeholders further endorsed the use of existing digital platforms, notably WhatsApp, as an alternative way to reach out to clients and provide support. Leveraging existing community support groups as an entry point for people in need of NCD care was highlighted as a win-win by all stakeholders. A final framework for an NCD care package supported by Friendship Bench was presented to policymakers and accepted to be piloted in five geographical areas.

**Conclusions:**

The ToC can be used to build consensus on how best to use using an existing intervention for common mental disorders to integrate care for diabetes and hypertension. There is a need to evaluate this new intervention through an adequately powered study.

**Supplementary Information:**

The online version contains supplementary material available at 10.1186/s12913-021-06957-5.

## Background

Non-communicable diseases (NCDs) are projected to become the leading cause of disability and mortality in sub-Saharan Africa by 2030, overtaking infectious diseases [1]. Recent research shows an exponential rise in diabetes, cardiovascular disease and mental ill-health in low- and middle-income countries (LMICs) [2]. For instance, a recent meta-analysis from Sub-Saharan Africa (SSA) reveals low detection and control rates for diabetes and hypertension [3, 4]. This low detection suggests a need for policymakers to consider diagnostic strategies to improve screening to optimise NCD care [3, 5]. Furthermore, a vast NCDs treatment gap exists. As much as 50 and 90 % of those in need of diabetes care or evidence-based mental healthcare, respectively, will not get it [4, 6].

A recent systematic review on priorities for disease management of NCDs in primary care identified the following as critical; availability of essential diagnostic tools and medications at local primary healthcare clinics and using standardised protocols for diagnosis, treatment, monitoring and referral to specialist care [7]. Also, task-shifting from physicians to non-specialist or community health workers, if accompanied by health system restructuring, is a cost-effective and sustainable strategy for improving NCDs care. [8]. Although task-shifting appears to be a rational approach, evidence suggests that if not done properly, it may bring about tension between existing professionals from whom tasks are shifted, resulting in fragmentation of care [9–11]. The tension comes as a result of role boundary disputes and professional resistance [9]. A theory of change can mitigate these challenges because it fosters a common understanding amongst all the stakeholders about what the intervention program is trying to achieve and how [12]. There are several examples of successful task-shifting models from sub-Saharan Africa, particularly in the HIV/AIDS field [13], with evidence showing that task-shifting models are generally both clinically- and cost-effective in low-resource settings [14]. Several studies have highlighted the feasibility of using non-professionals through task-shifting to manage a range of NCDs [8]. However, there is a dearth of information on how a joint task-shifting approach for several NCDs, namely diabetes, hypertension and common mental disorders, can be designed.

In Zimbabwe, all three NCDs are managed at the primary health care level by different health care workers. Each condition is assigned a specific consultation day during the week, resulting in multiple clinic visits for clients with NCD comorbidities. There is also a paucity of research on task-shifting in diabetes and hypertension in Zimbabwe. However, robust evidence on task-shifting for common mental disorders exists through the Friendship Bench [15]. The Friendship Bench (FB) is a task-shifted, cost-effective and brief psychological intervention delivered by trained community grandmothers for common mental disorders (CMDs) such as depression and anxiety [15, 16]. The Friendship Bench intervention (FB) is based on cognitive behaviour therapy principles with an emphasis on problem-solving therapy (PST) [16]. The Friendship Bench intervention was developed in response to the mental health treatment gap in Zimbabwe. In Zimbabwe, mental health services are chronically underfunded, which is exacerbated by a massive shortage of trained mental healthcare practitioners [15, 16]. To date, over 500 grandmothers have been trained in the last 10 years to deliver the Friendship Bench on wooden park benches across 100 communities. Anecdotal evidence based on over 200 weekly de-brief sessions with the grandmothers suggests that the grandmothers often encounter clients with a combination of depression and comorbid diabetes and hypertension. Substantial evidence supports the high comorbidity of the three conditions globally [17–19]. This paper describes the process of developing an integrated intervention for depression, diabetes and hypertension using a theory of change approach.

## Methods

As part of a study aimed at integrating diabetes and hypertension care into the Friendship Bench, we carried out a theory of change (ToC) workshop with key stakeholders to build consensus and define the pathway to integrating diabetes and hypertension care into the Friendship Bench CMD care package. A ToC is a structured approach of bringing together different stakeholders to build consensus on a common initiative. We have used this approach extensively to build consensus in scaling up the Friendship Bench in Zimbabwe [20]. We opted for the ToC approach because of the different competing needs for the three NCDs and the need to build consensus on leveraging lessons from the Friendship Bench to integrate all three conditions into a single package of care. We sought to use the ToC to build consensus among stakeholders on how best to develop a care package for the three NCDs.

### Setting

We initially carried out a situational analysis of the management of the three NCDs in primary care facilities in the country (results are reported elsewhere). Following the situational analysis, a survey in five (5) primary care facilities looking at the burden of the NCDs was carried out; again, this data is described elsewhere. Also, we carried out semi-structured interviews with a wide range of stakeholders to appraise the current NCD management practices, including exploring the barriers and facilitators of the management of depression, diabetes and hypertension in primary care facilities in Zimbabwe. Findings from the situational analysis, survey and qualitative interviews were shared with key stakeholders before the ToC workshop.

### Participants

We invited 20 participants to attend the ToC, with each participant provided with a detailed background to earlier work before the ToC. Eighteen participants attended the ToC, and these included; four (4) researchers and clinicians from the Friendship Bench, lay health workers (*n* = 4), persons with lived experience (i.e. people living with NCDs) (*n* = 4), Zimbabwe Diabetes Association Administrator (*n* = 1), a senior officer from the Zimbabwean Ministry of Health Policy Department (*n* = 2), and traditional healers (*n* = 2).

### Theory of change procedure

An appointed facilitator with experience facilitating ToC initiated the workshop by summarising the survey and situational analysis findings. This was followed by a group discussion of findings with the 18 stakeholders as the facilitator developed the ToC map through a co-production process. We used the checklist for reporting ToC in public health intervention as recommended by Breuer [21] to ensure that all the relevant components of our ToC were captured. The checklist covers five domains, the definition of ToC; the description of the ToC development process; the ToC diagram; the process of intervention development and the use of ToC in evaluation.

## Results

**Fig. 1:**
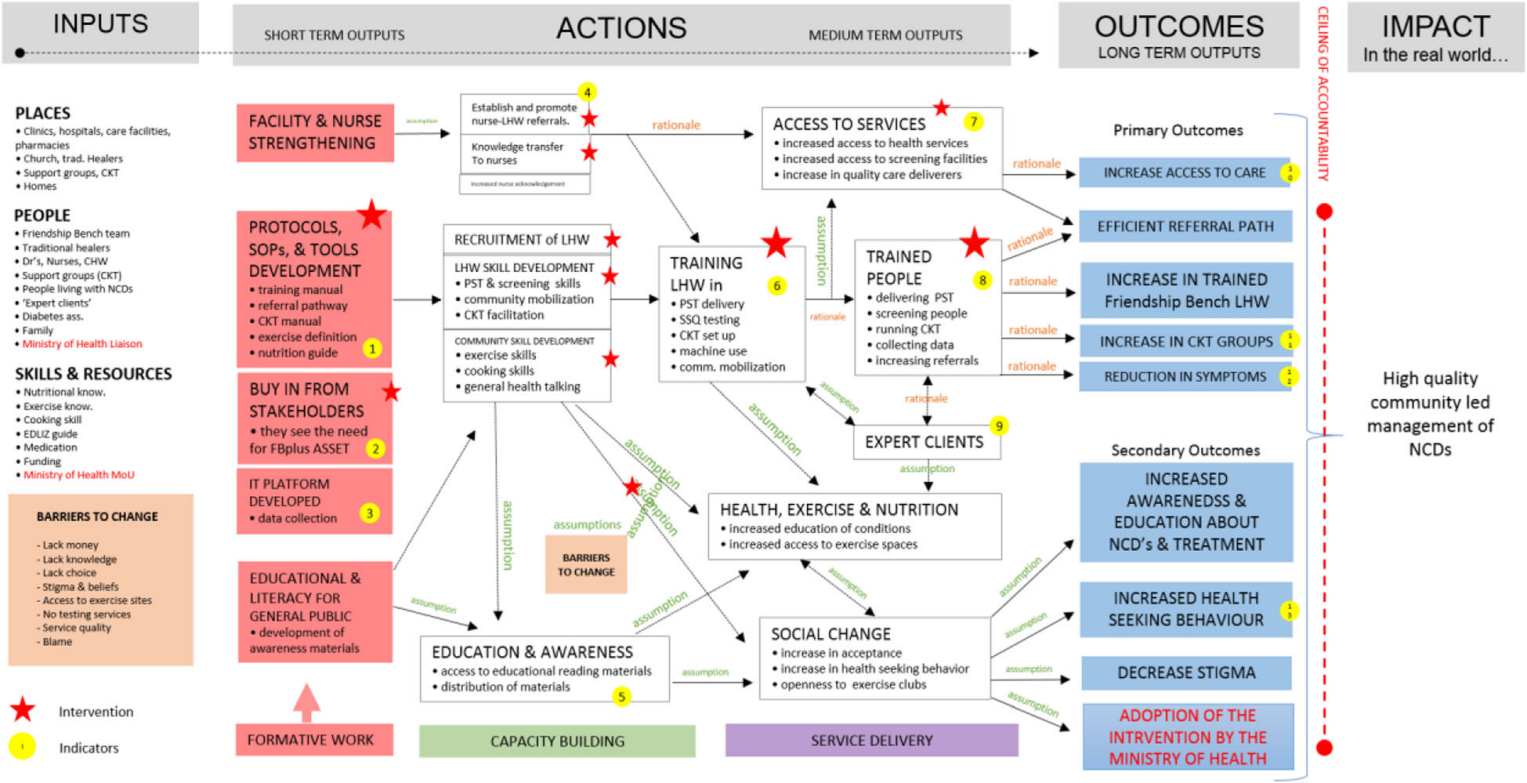
Theory of change map for integrating 3 non-communicable disease based on Friendship Bench model. Key: ASSET -Health System Strengthening in Sub Saharan Africa; CHW- community health worker; CKT- Circle Kubatana Tose; EDLIZ- Essential Medicines List and Standard Guidelines for Zimbabwe; FBplus- Friendship Bench Plus; LHW- lay health workers; MoU- Memorandum of understanding; NCD-non-communicable disease(s); PST- problem-solving therapy; SSQ- Shona Symptoms Questionnaire

There was an initial consensus on the need to have high-quality, community-driven management of NCDs, which were context-specific. The Zimbabwean Ministry of Health was particularly keen to explore how a recently signed memorandum of understanding (MoU) with the Friendship Bench could be used as leverage to address broader NCD issues using task-shifting. For all three NCDs, the following overarching desired outcomes for an integrated package evolved; a need to increase access to person-centred care, efficient referral systems, and improved health-seeking behaviour(s) (See Fig. 1). After defining the ceiling of accountability and outcomes of the initiative, the ToC facilitator led the group to identify existing resources for optimal implementation. The grandmothers highlighted the context they were providing services for the three NCDs with very little structure offered for diabetes and hypertension and emphasised the need for some structure to target diabetes and hypertension in the same way that they used the PST approach for CMDs. During the deliberations, it was agreed to place resources into three categories, i.e., ‘Skills and Resources’, ‘People’ and ‘Places’ (Fig. 1). In the short term, it was agreed that facility, nurse and grandmother strengthening would be critical to ensure that the capacity to manage an integrated NCD package was in place with a clear referral pathway. This was seen as an important intervention with a consensus of key output/indicator being the number of people successfully completing training and showing adequate knowledge to provide services for the three NCDs. In the long term, there was consensus on the need to develop an intervention that could be assimilated into primary health care policy in the same way that the original Friendship Bench model was integrated into primary health care policy. This, it was agreed, would allow the intervention to be scaled up nationwide and leverage the MOU that already exists between the Friendship Bench and the Ministry of Health. Other components of the intervention with corresponding indicators are provided in the ToC map (Fig. 1).

### Friendship Bench resources

Social media was identified by the grandmothers, nurses and people living with NCDs as a low-hanging strategy for strengthening NCDs services provision in the face of the COVID-19 pandemic. The WhatsApp platform was identified as easily accessible for communication and dissemination of knowledge, patient referrals, and simple advice. Sharing short voice messages with clients via WhatsApp was particularly appreciated by the grandmothers to deal with the COVID-19-related lockdown measures. The grandmothers and people living with NCDs felt that integrating WhatsApp messaging for all three NCDs would be feasible. Other existing Friendship Bench structures that were identified during the ToC included the community support groups, locally referred to as circle kubatana tose (CKT), which were linked to health facilities., the screening tools for CMD, the SSQ-14, which could be used to integrate questions linked to diabetes and hypertensionThere was consensus between the grandmothers and people with lived experience on the negative effects of low health literacy; communities had low awareness of preventive measures for hypertension and diabetes. Also, people with lived experiences attending the ToC highlighted the need to use “expert clients” to ensure that a person-centred approach to the care of the NCDs was considered. Expert clients can be defined as people living with a long-term health condition who have significant knowledge of their condition and treatment who can manage their conditions, thus leading to an improved quality of life [22]. It was suggested that expert clients could be linked to the grandmothers through the community support group (CKT), which in some communities had been running for close to 10 years, and have the primary care nurse provide support and review of all those who were referred to the clinic from the community for the three NCDs.

**Fig. 2:**
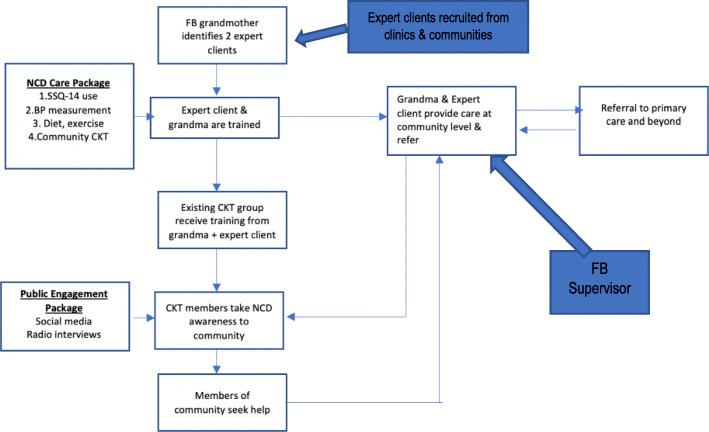
NCD Friendship Bench intervention based on theory of change map. Key: CKT-Circle Kubatana Tose; FB- Friendship Bench; NCD- non-communicable disease(s); SSQ-14: Shona Symptoms Questionnaire-14

Based on the ToC findings, a smaller group consisting of Friendship Bench team members integrated vital findings from the ToC to develop a framework for a draft intervention and its components (Fig. 2). We used Escoffery’s framework on the adaptation of existing evidence-based interventions (EBI), which emphasise community assessment, understanding the existing EBI, consulting stakeholders, agreeing on adaptations and adapting the original intervention to guide the process [23]. Furthermore, during the ToC, themes that appeared to be critical for stakeholders, which included the context, implementation and setting of the adopted Friendship Bench for NCDs, subsequently informed the selection of the Context and Implementation of Complex Interventions (CICI) framework [24] for the evaluation of the intervention. The CICI framework is a determinant and evaluation framework that comprises three dimensions- context, implementation and setting that interact with one another and the intervention at a micro, meso and macro level. Context focuses on seven domains (i.e., geographical, socio-cultural, epidemiological, legal, ethical and political). Implementation has five (implementation theory, strategies process, agents and outcomes) while setting refers to the actual physical location where the intervention is implemented [21].

### Intervention

The existing Friendship Bench structures, which had been running for over 10 years and recently supported through an MoU with the Ministry of Health and Child Care (Zimbabwe), would form the basis for the identification of expert clients who would go on to work with the grandmothers and primary health care nurses. Grandmothers would be paired with expert clients and be trained in NCD care based on an NCD care package/algorithm developed from the situational analysis, survey of five primary health care facilities and a series of qualitative interviews (all described elsewhere). A public engagement strategy based on consensus from the ToC and leveraging Friendship Bench existing resources (www.friendshipbenchzimbabwe.org) would be launched before the pilot of the NCD package. The trained grandmothers and expert clients would provide training to existing CKT community groups. Members of these groups would then disseminate information about the three NCDs in their communities, resulting in community members seeking services from the grandmother and expert client (Fig. 2).

## Discussion

There is growing evidence showing the widespread use of the theory of change (ToC) to develop and evaluate public health interventions [21]. Such a theory-driven approach improves the design and evaluation of complex interventions, thereby increasing the likelihood that the intervention will be ultimately effective, sustainable and scalable [25]. Although we have previously used the ToC to design and evaluate a complex intervention, the Friendship Bench [15, 20], this is the first time a theory of change (ToC) has been used in SSA as a tool to integrate hypertension and diabetes care into an existing evidence-based care package for common mental disorders delivered by trained community health workers. Our earlier work has shown the effectiveness of using grandmothers (community health workers) to deliver evidence-based therapy for common mental disorders in a low resourced setting [15]. A critical feature of our work involved the inclusion of these local and experienced grandmothers and people with lived experience to co-create the intervention. This approach was influenced by our earlier experience of using the ToC to build consensus on key intervention components for the Friendship Bench [20]. For instance, both the grandmothers running Friendship Bench and people with lived experience attending the ToC believed using a similar approach based on opening the mind “kuvhura pfungwa” [26] could successfully address issues and suggest strategies around hypertension such as reducing salt intake and increase physical exercising which could be included in the existing FB services. Similarly, it was felt that including key questions to inform both grandmothers and the communities on diabetes was possible using this approach. Therefore, an important feature of this work was the ToCs ability to leverage existing acceptable community idioms of illness to integrate hypertension and diabetes care into the Friendship Bench [26].

Both hypertension and diabetes are increasingly recognised as the leading causes of disability in SSA, needing urgent addressing [2]. While a recent review of interventions for NCD’s delivered by CHWs suggests that, compared with standard care, engaging CHWs in health programmes have the potential to be effective in LMICs, particularly for tobacco cessation, blood pressure and diabetes control [27], there remains a dearth of knowledge on how best to integrate multi-component interventions to address several risk factors concurrently. In mental healthcare, transdiagnostic approaches that provide cross-cutting treatments for many conditions have been successful, particularly for common mental disorders such as anxiety, depression, and other affective conditions [25, 28]. While a recent review supports this approach for hypertension and diabetes, through interventions offered by CHWs, which included health education/health promotion (lifestyle modification advice) for diabetes, cancer, cardiovascular diseases and stroke prevention [27], it did not mention interventions that also incorporate mental, neurological and substance use disorders (MNS) which are among a growing problem in LMIC [29]. There is, therefore, a paucity of data on how best to integrate care for hypertension and diabetes into an existing mental health intervention that focuses on CMDs.

Comorbidity between CMDs, hypertension and or diabetes is common and untreated; CMDs can worsen disease outcomes for both hypertension [30] and diabetes [31], with earlier research showing that anxiety and depression are predictive of later incidence of hypertension [32] and diabetes [33]. Based on the increasing evidence for the effectiveness of CHWs’ ability to manage a wide range of NCDs [8, 34], we have used a ToC approach to build consensus on the first integrated care package for three NCDs. The framework described above (Fig. 2) will be piloted in two sites before it is adjusted and re-piloted. As in all our previous intervention developments, we will rely heavily on community involvement in the co-creation of the final package.

What strengthens our approach is using existing structures through the Friendship Bench and the formal support by the government of Zimbabwe through an MoU that seeks to take to take scale this approach to narrow the treatment gap for the three conditions. The previous Friendship Bench ToC map involved community health workers trained in problem-solving therapy and providing counselling services to primary care attendees. Like the current ToC map, it highlighted the need for political buy-in and capacity-building, particularly for the community health workers and developing a feasible and user-friendly psychological intervention. The difference between the two maps is that the new map incorporates hypertension and diabetes care into an existing evidence-based psychological intervention. The use of the grandmothers, particularly those who have been involved with Friendship Bench for over 10 years together with expert clients, provides a strong possibility of sustainability. The consensus between the grandmothers and clients about the negative effects of poor general health literacy has been shown in an early systematic review [35, 36].

To create a more dynamic model the theory of change is going to be augmented by a series of qualitative studies (i.e. focus group discussions, interviews). Complex interventions typically seek to change the social system as such dynamic logic models have feedback loops that provide an opportunity to adapt the intervention to suit its context, potentially changing the intervention strategies and the outcome produced [37]. The theory of change will go through an iterative adaptation process that involves an analysis of qualitative data collected throughout the project. The iterative refinement will make it possible for the model contents to be tested and refined as new information comes to light and themes emerge. This will be done inductively or combine deductive elements with prior theory informing the initial contents of the theory of change.

Despite these key strengths, major limitations include the possibility of over-burdening the grandmothers, of whom some are already struggling with managing clients on the Friendship Bench. However, as highlighted in the ToC, the grandmothers acknowledged dealing with clients with diabetes and/or hypertension and desire to have some structured approach mainly through the involvement of the expert client and stronger presence of the community through the support groups. It is, therefore, possible that this integrated care package could result in the alleviation of work pressure for the grandmothers. Also, the current COVID-19 pandemic poses logistical challenges in implementing the proposed study. For example, it is uncertain how long it would take to implement key arms of the intervention, such as identifying expert clients. Also, as much as there is high mobile penetration in Zimbabwe, the potential impact of high mobile data costs on implementing the proposed intervention is unknown.

## Supplementary Information

**Additional file 1.**

## Data Availability

The data that support the findings of this study are available from the corresponding author upon reasonable request.

## Abbreviations

CHW: Community Health Worker
CKT: Circle Kubatana Tose
CMD: Common Mental Disorders
FB: Friendship Bench
HIV/AIDS: Human Immune Virus/Acquired Immune Deficiency Syndrome
LMIC: Low- and middle-income countries
MoU: Memorandum of understanding
NCD: non-communicable diseases
SSA: Sub-Saharan Africa
SSQ14: Shona Symptoms Questionnaire 14
ToC: Theory of Change
