# Supplementary material for: Using a theory of change to develop an integrated intervention for depression, diabetes and hypertension in Zimbabwe: lessons from the Friendship Bench project
Source: BMC Health Serv Res. 2021 Sep 7;21:928. doi: 10.1186/s12913-021-06957-5 (PMC8421086; doi:10.1186/s12913-021-06957-5)
Supplement: Supplementary file 1 — Additional file 1. [file 12913_2021_6957_MOESM1_ESM.docx]

**Situational Analysis**: Using a theory of change to develop an integrated intervention for Depression, Diabetes and Hypertension in Zimbabwe: Lessons from the Friendship Bench project

| Study Investigators | Professor Dixon Chibanda & Dr Ruth Verhey |
| --- | --- |
| Year of study | 2018-2021 |
| Country | Zimbabwe |
| Place in country | Harare |
| Urban Rural or Mixed | Urban |
| Objective | To investigate the burden of non-communicable diseases in primary care clinic |
| Study Population | Health workers and clinic attendees |
| Methods used | Survey, semi structured interviews, Focus group discussions |
| Key Findings | **Quantitative findings**  The survey of 1,240 unselected primary healthcare attendees in six primary healthcare centres revealed that 84% were female. We found a high prevalence of hypertension (39%), diabetes (7%), depression (11%) and HIV (45%). Multimorbidity was present in 23% of attendees. Problem drinking was identified in 26% of male and 3% of female attendees. Obesity was found in 42% of men and 8% of women. 19% of men and 4% of women were current smokers.  **Semi Structured interviews**  Barriers to care at primary care level include   - Unavailability of Medication - Shortage of equipment/machinery - Lack of human resource - Poor Remuneration - Poor community awareness of NCDs   Patients not coming to the clinic on time or at all  **Focus Group Discussions**   - Community Health workers (CHWs) were already theoretically mandated to conduct NCD health promotion and prevention activities in the community, but lacked materials, training and skills. - Nurses from PHC facilities were also supposed to provide community outreach, but this activity had dwindled. - There were limited opportunities for interaction and interactivity between CHWs and PHC facility-based teams, particularly around referral from the community to the facility, and vice versa. - There is a need to deliver health promotion and prevention advice in the community. |
| Socio-economic and behavioural drivers | Lack of knowledge on NCDs, poverty, national economic decline, shortage of medication and equipment at the clinics |
| Output/ Impact | N/A |
| Limitations | Non cited |
| Implication for future programs | There is a need to leverage and strengthen existing community substructures to improve equitable access to basic health promotion and NCD prevention services, and better-quality continuing care for those who need it.  There is a need for task-shifting basic non communicable diseases from nurses to community health workers to reduce their workload.  There is also a need for continual lobbying to policy makers to improve working conditions and supply basic clinical consumables and essential drugs through a holistic and multisectoral approach. |
| Citations | Non cited. |
